# Social Determinants of Inter-Individual Variability and Vulnerability: The Role of Dopamine

**DOI:** 10.3389/fnbeh.2022.836343

**Published:** 2022-03-21

**Authors:** Philippe Faure, Sophie L. Fayad, Clément Solié, Lauren M. Reynolds

**Affiliations:** Brain Plasticity Laboratory, CNRS UMR 8249, ESPCI Paris, PSL Research University, Paris, France

**Keywords:** dopamine, variability – individual, vulnerability, social behavior, micro-society, ethological analysis

## Abstract

Individuals differ in their traits and preferences, which shape their interactions, their prospects for survival and their susceptibility to diseases. These correlations are well documented, yet the neurophysiological mechanisms underlying the emergence of distinct personalities and their relation to vulnerability to diseases are poorly understood. Social ties, in particular, are thought to be major modulators of personality traits and psychiatric vulnerability, yet the majority of neuroscience studies are performed on rodents in socially impoverished conditions. Rodent micro-society paradigms are therefore key experimental paradigms to understand how social life generates diversity by shaping individual traits. Dopamine circuitry is implicated at the interface between social life experiences, the expression of essential traits, and the emergence of pathologies, thus proving a possible mechanism to link these three concepts at a neuromodulatory level. Evaluating inter-individual variability in automated social testing environments shows great promise for improving our understanding of the link between social life, personality, and precision psychiatry – as well as elucidating the underlying neurophysiological mechanisms.

## Introduction

Inter-individual variability refers to differences in the expression of one or more behaviors between members of a population. For instance, some people express a shyer attitude than others, take more risks, or are more attracted to immediate gains. This variability is also evident in the way one responds to environmental and social challenges, resulting in a heterogeneous expression of emotional, cognitive, and task-related behaviors; and underlies, in particular, the emergence of distinct strategic approaches (i.e., how agents find different solutions to the same problem). In human studies, such behavioral variations have long been associated with the notion of personality (McAdams and Pals, 2006). In animal research, however, behavioral variability has largely been considered as unwanted noise, or as an experimental confound, and thus disregarded. But the consistency of these inter-individual differences across time and contexts has become harder to overlook, and it is now generally acknowledged that animal personalities are ubiquitous, quantifiable, and biologically meaningful (Sih et al., 2004; Bach, 2009; Bergmüller and Taborsky, 2010; Duckworth, 2010; Pennisi, 2016).

While the concept of personality in animals is now increasingly accepted, the mechanisms underlying the generation of inter-individual variability are still poorly understood and a major current topic in adaptive personality research (Sih et al., 2015). Ecologists have framed the significance of this process from a genetic point of view, proposing that the mechanisms driving individual variability may play a role in evolution by helping segregate species into subpopulations (Pennisi, 2016). However, many teams have observed that even under controlled laboratory conditions, behavioral expression varies much more than expected between virtually genetically identical individuals (Buchanan et al., 2015; Stern et al., 2017; Tuttle et al., 2018), suggesting a key role of the environment in driving individuation processes (Lathe, 2004; Stamps and Groothuis, 2010). Behavioral differences between individuals have been linked with variance in their physiology [e.g., body size, metabolism, neurophysiological properties (Dingemanse and Wolf, 2010)], in local environmental factors (particularly the distribution of resources, such as food, shelter, and breeding opportunities), and in their life history. The latter critically relies on brain plasticity properties, which encode an individual’s experiences to shape their response to upcoming environmental challenges in a cumulative manner, thus supporting the behavioral divergence of initially genetically identical mice (Freund et al., 2013). Another point of view is that individuality is an unpredictable outcome of developmental processes (Stern et al., 2017; Wolf et al., 2017; Honegger and de Bivort, 2018). In this stochastic developmental variability hypothesis, individuation results from the accumulation of differences during development that, in turn, generate structural variations in neural connectivity patterns and capacity for plasticity, which then remain stable through adult life (Buchanan et al., 2015; Stern et al., 2017; Wolf et al., 2017; Honegger and de Bivort, 2018). This view is consistent with the definition of individuality as characteristic behavioral traits that persist over a lifetime (Honegger and de Bivort, 2018).

These alternative perspectives on inter-individual variability do not necessarily contradict each other, instead they highlight that distinct forms of individual adaptation or plasticity may operate over different time scales. The influence of the environment on the development of inter-individual variability and personality is most often discussed in terms of a developmental process. In this review we focus instead on the highly dynamic individuation processes that occur across the lifetime as an adaptation to proximal environments, and in response to social interactions in particular. We define adaptation as an animal’s flexible adjustment of their behavior over time, in response to situations and by using the cumulative knowledge of their previous experiences. The role of underlying neural components in individuation has been framed in terms of continued developmental processes [e.g., adult hippocampal neurogenesis (Kempermann, 2019)]. Here, we examine the growing evidence that changes in the activity of neuromodulatory networks link social influences with adaptations to egocentric (i.e., non-social) behaviors in adult animals. While multiple neuromodulators are likely involved, we focus here on modifications in dopaminergic circuits, which have been strongly linked to the individualistic expression of exploration behavior. Finally, we discuss how these views, in which circuits are changed through adaptation, can improve our understanding of the link between behavioral trait expression and vulnerability or resilience to psychiatric illness. Each of these aspects will be explored from the perspective of rodent micro-society behavioral paradigms, which are generally large, controlled environments where rodents live in groups (their “micro-society”) with automated capture of behavioral information over long periods of time. These testing environments are increasingly developed in neuroscience research laboratories and provide exceptional insight into both naturalistic social interactions and inter-individual behavioral variability.

## Defining and Measuring Inter-Individual Variability: From Experimental Confound to Experimental Outcome

**FIGURE 1:**
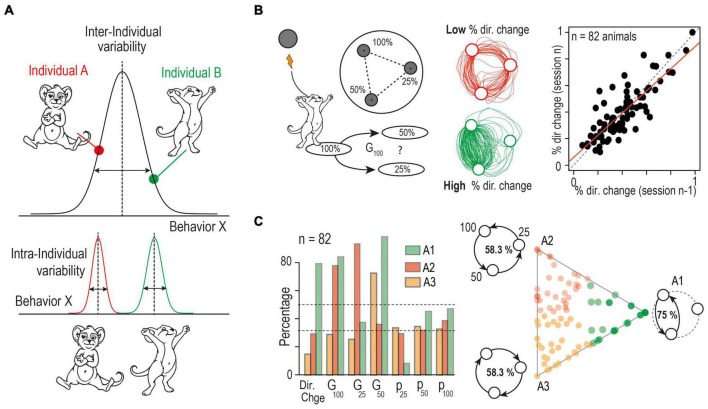
Theoretical framing and experimental distinction between inter- and intra- individual variability. **(A)** Defining intra and inter-individual variability: the frequency and intensity with which an individual exhibits a specific behavior defines a trait. The estimate of the trait expression at the population level defines inter-individual variations. Repeated measurements on individual subjects allows the estimation of intra-individual variations in trait expression. **(B)** (*Left*) Schematic of a behavioral decision-making paradigm where inter- and intra-individual variability is apparent. Mice are placed in a circular open-field with three equidistant targets that are associated with a given probability (100, 50 or 25%) of intracranial stimulation reward delivery when the animal is detected around the target. The animal cannot receive two consecutive stimulations from the same target; thus they must make a sequence of binary choices between the targets. The trajectories and choice or each mouse are quantified using, (i) the percentage of directional change (i.e., returning to the previous target, % dir. change), (ii) the probability to visit each point (P_100_, P_50_, P_25_) and (iii) the probability to choose the option with the highest probability of reward for the three possible “gambles”: G_100_ = choice of 50% over 25%, G_25_ = choice of 100% over 50 and G_50_ = choice of 100% over 25%. (*Center*) Example of two trajectories obtained from two different mice across a 5 min session showing individualistic strategies where the mouse in the top trace shows a low propensity for changing direction, resulting in a more circular trajectory, while the mouse in the bottom trace shows a high percentage of directional change, focusing on the most rewarded options. (*Right*) Analysis over concurrent sessions indicates consistent differences in behaviors between mice indicative of low intra-individual variability and high inter-individual variability. Correlation between the percentage of directional changes for two consecutive sessions shows a strong stability between strategy across sessions within individual mice, while behavior between subjects remains variable. **(C)** Archetypal analysis: (*left*) Plot of the three archetypal solutions, and their seven basic variables (see **B**). *Left*: Visualization of the α coefficients using a ternary plot. Each point represents the projection of an individual onto the plane defined by a triangle where the three apices represent the three archetypes (A1, A2, A3). Points are color-coded according to their proximity to the archetypes. Schematic at the three apices illustrate the main behavior of the three archetypes. A1 corresponds to a mouse that alternates only between p_100_ and p_50_. Such a mouse would reach a 75% success rate. In contrast, mice using a purely circular strategy would have a 58.3% success rate. They either turn in a descending manner (A2: sequence p_100_ – p_50_ – p_25_) or an ascending manner (A3: sequence p_25_ – p_50_ – p_100_). Experimental mice fall somewhere within the boundaries of these extreme behaviors. (Modified from Dongelmans et al., 2021).

It is easy to recognize qualitatively and anecdotally that each individual is unique in the way it behaves, an idea that incorporates two seemingly contradictory quantitative aspects of behavioral variation. On one hand, intra-individual variation encompasses the diversity in behaviors and actions that occur within the same individual over time and context (for example, an individual facing a binary choice may once choose the first option and another time the second one, or *vice versa*). On the other hand, inter-individual variability can be conceptualized in some ways as the invariability of behaviors. For example, when repeatedly faced with a binary choice, some individuals choose the first option 80% of the time on average, while others will choose the first option on average only 40% of the time. In this sense, inter-individual variation produces a stable behavioral repertoire that characterizes an individual and distinguishes it from its conspecifics (Figure 1A). This idea has consequences regarding how behavior is analyzed, as the bulk of behavioral experiments have been designed in ways that ignore or minimize inter-individual variability, stemming both from conceptual limitations and from technical constraints. We argue that, instead, acknowledging and assessing inter-individual variability can clarify the relationships between brain and behavior, as well as between behavioral adaptation and variation. Incorporating measurements of inter-individual variability in behavioral outcomes can be simplified by using large, automated testing environments, such as those that support the study of rodent micro-societies, thus we also discuss some of the advantages and challenges that these environments provide.

The distinction between inter- and intra-individual variability goes against traditional behavioral analysis framework that uses the behavior of the group to derive an average “individual”, and to establish the standard deviation from this norm (Bennet, 1987). In this approach, one considers that the information accumulated about populations applies uniformly to their constituent individuals: in other words, sampling a behavior across multiple subjects at the same timepoint would be conceptually the same as using repeated measurements on a single subject, thus there is no need to distinguish between inter- and intra-individual variability. This approach has been heavily exploited to allow meaningful between-group comparisons, particularly in animal research where individuals can indeed largely be considered as identical except when specific conditions are manipulated (e.g., environmental or genetic modifications). However, by assuming that each subject can be described by the behavior of the group, this approach masks the different contributions of intra- and inter-individual variability to overall phenotypic variability. To reveal their balance, one needs to explicitly compare intra- and inter-individual variances by performing repeated measurements on each subject within a testing group (Figure 1B). If the multiple expressions of a behavior in the same individual follow their own distribution, the estimates of the inter-(V_inter_) and intra-individual (V_intra_) variances will differ, with V_inter_ being greater than V_intra_. Efforts to standardize these methods to improve study design and interpretation have yielded several measures. For example, the repeatability index (R) has been proposed as a standardized measurement of phenotype consistency across time or contexts. It corresponds to the proportion of the total phenotypic variance [defined as the sum of the inter-individual (V_inter_) and intra-individual (V_intra_) variances] that can be attributed to inter-individual variance: R = V_inter_/(V_inter_ + V_intra_) (Nakagawa and Schielzeth, 2010; Roche et al., 2016). Similarly, analyzing the cumulative value of an estimator over a long-term experiment [e.g., an estimator of exploration level, Freund et al. (2013), Torquet et al. (2018)] highlights the coherence in behavioral differences between individuals across time. For example, a mouse with a low exploration level may increase it over time, but it will typically remain at a lower level than its congeners. Overall, these approaches demonstrate that intra-individual variation is generally smaller than inter-individual variation, indicating consistency over time in the behavior of individuals, and arguing against “population” assumption in behavioral experiments. Indeed, by defining stable behavioral traits in an individual, relationships can be revealed between the expression of this trait and of other behaviors, physiological characteristics, or brain activity; which may otherwise have remained hidden in a purely group-based analysis.

Time scale is another important consideration for distinguishing between inter- and intra-individual variability in behavioral testing and analysis. Classic tests used to measure individual characteristics or traits often focus on specific behaviors only observed over a short time scale, generally on the order of minutes. Appropriately distinguishing between inter- and intra-individual variability requires instead the generation of repeated measurements over long time frames, on the order of weeks to months. This allows the accumulation of information regarding how an individual behaves in response to the same stimuli over time, which allows not only the estimation of intra-individual variability, but to also estimate inter-individual variations in behaviors and establish individualistic profiles. These longitudinal experiments necessitate the transition to automatic testing and processing, which is already supported in principle and/or in practice by a number of neurobiologists (Dell’Omo et al., 2000; Gerlai, 2002; Vyssotski et al., 2002; Spruijt and DeVisser, 2006; Sandi, 2008; Schaefer and Claridge-Chang, 2012). This idea has led to the development of complex housing environments for laboratory mice (Freund et al., 2013; Shemesh et al., 2013; Weissbrod et al., 2013; Torquet et al., 2018) that allow the integration of automated behavioral testing. These apparatuses engender several significant advantages over traditional testing methods: rodent behaviors can be evaluated without isolating an individual from its social group, measurements for several behavioral parameters can be simultaneously captured, and *post hoc* analyses of behavioral correlations can be used to construct an individual estimator defining each subject (Freund et al., 2013; Torquet et al., 2018; Forkosh et al., 2019).

Longitudinal video tracking of animals over long periods presents its own challenges, namely definitively identifying each individual and correctly assigning their behavioral variables. A single case of mistaken identity would call into question the validity of all the results acquired from months of work. Different solutions to this problem have been proposed. One of them is to dye the fur of the animals with different colors (Shemesh et al., 2013; Forkosh et al., 2019). Another one is to implant radio-frequency identification (RFID) transponders under the skin of the mice to assign an ID number to each subject, while detectors built into the environment can track the identity of mice and confirm or correct video tracking (de Chaumont et al., 2019). Many systems are now able to evaluate the specific postures of individual (e.g., locomotion, self-grooming) or interactive behaviors between two or more identified individuals [e.g., nose to nose contact, playing, aggression, peer grooming, de Chaumont et al. (2012, 2019), Mathis et al. (2018)]. On-line position tracking or *post hoc* pose estimation overcome traditional challenges that result from relying on observer scoring to establish and analyze behavioral patterns. Advances in these technologies are poised to drive the implementation of automated and standardized analysis of behavioral repertoires, which are holistic compilations of behaviors described by an observer, and can be considered to be built at their most basic level from positional changes of an animal over time.

While these automated testing environments generate large data sets, classification and dimension reduction methods can be used to compact this information in order to isolate behavioral domains and to establish correlations between them (Brown and de Bivort, 2018). Clustering methods such as *k-means* or principal component analysis are the most commonly used to discriminate average behaviors, where an individual’s distance to each cluster describes the relationship of its behavior to that of its congeners. While these methods have been classically used to aggregate individual data onto typical observations represented by the center of a cluster, they are not the only approaches to analyze behavioral repertoires: archetypal analysis depicts individual behavior instead as a continuum within an “archetypal landscape” defined by “pure” or “archetypal” behavioral patterns. With this method, the most extreme or specialized behavioral profiles possible from the entire data set are first defined as the archetypes. The number of archetypes and their associated behavioral patterns are derived from the dataset in an unsupervised manner, and each individual’s behavior can then be described as a convex mixture of archetypal profiles (Cutler and Breiman, 1994; Shoval et al., 2012; Forkosh et al., 2019; Dongelmans et al., 2021). The individuals can be assigned to the archetype that best describes their behavior for experimental grouping purposes, rather than defining groups by an arbitrary threshold on any one continuous variable. For example, strong and stable individual strategies emerge in a decision-making task where mice are required to move between three sites with different probabilities to receive rewarding electrical stimulation (Figure 1B). Archetypal analysis uses the key choice parameters from the task to reveal the three most extreme possible strategies: alternating exclusively between the two options with the highest probability of reward (A1), purely traveling in a circular pattern moving from the highest to lowest probability of reward (A2), or from the lowest to highest probability (A3). Therefore, individual behaviors find themselves somewhere between these extreme strategies, and can be defined as a linear combination of each archetype (Figure 1C; Dongelmans et al., 2021). These approaches have important consequences for introducing the notion of “personalized” behavioral assessment: by allowing the dissection of the contribution of inter- and intra-individual variability to phenotypic variability, they challenge classic approaches based on the analysis of average group behavior measured at a given moment.

Finally, the implementation of these semi-natural and social testing environments increases the complexity of the research questions that can be addressed, in particular raising questions about (i) how these environments promote the emergence of individual behavioral variability (Kempermann, 2019), and (ii) how animals living in a micro-society deal with complex and ethologically valid decision-making problems. These problems are defined by the particular conditions of their habitat, notably the food distribution and the social milieu (Dell’Omo et al., 2000; Mobbs et al., 2018). Foraging for food is, for instance, a very important aspect of animal life, and represents one of the basic mechanisms studied in neuroeconomics (Hayden and Walton, 2014; Hayden, 2018; Mobbs et al., 2018). In such closed-economy paradigms, commodities (food or other rewards) are present at all times, in contrast to standard laboratory tests. Thus, the initiation and termination of consummatory behaviors are defined solely by the animal (Timberlake and Peden, 1987; Rowland et al., 2008; Beeler et al., 2010), which significantly modifies our conceptual framing of reward studies. In these environments the dependent variables are rather defined by the sequence of reward related-behavior and the amount of time budgeted by the animal for each of its activities, than by the amount of reward earned by an individual in a restricted amount of time. Social interactions will also constrain the expression of foraging behaviors. An isolated animal must invest time and resources to explore and search for food, while being part of a group may open new opportunities or responsibilities. On the one hand, an individual within a group, may be able to wait for others to find food, on the other hand the group could instead exploit this individual, redistributing the food it has foraged for itself among group members (Barnard and Sibly, 1981; Giraldeau and Dubois, 2008). Overall, foraging for food drives the development of a large number of social interactions, whether cooperative or exploitative, and promotes the development of individual strategies (discussed in detail below).

Once considered nothing more than noise, inter-individual variability is increasingly considered measurable and meaningful, particularly thanks to conceptual and technical advances in behavioral data collection and analysis. The adoption of large, automated testing environments allows the tracking of individual mouse behavior within a micro-society living in a complex environment over long periods of time. In that context, both the processes operating within individuals as well as those operating between individuals at the population level can be described (Giraldeau and Dubois, 2008). These advances are driving new perspectives in understanding behavior and its relationship to underlying neurocircuitry.

## The Role of the Social Environment in the Definition of an Individual

Standard laboratory housing consists in relatively impoverished environments that significantly restrict social contact, housing rats most often in pairs and mice in small groups of up to four congeners (Würbel, 2001). Rodents are, however, social animals; this is aptly evidenced by their repertoire of various interactive behaviors – such as physical contact, vocal communication, aggression, social recognition, imitation, and empathy – that can be considered as hallmarks of sociability, an important personality trait (Gartland et al., 2021). In the wild, mice live in small breeding subpopulations (demes) of 2 to 12 adult members (Crowcroft, 1966; Berry and Bronson, 1992; Beery and Kaufer, 2015) that share territorial defense, while rats generally live in larger colonies that may be divided into smaller sub-groups as a function of resources (Beery and Kaufer, 2015; Schweinfurth, 2020). The structure of rodent groups is highly malleable, with both the size and membership liable to change with resource availability, social competition, or death from predation or disease (Radchuk et al., 2016; Andreassen et al., 2021). The social environment of a rodent is therefore in constant evolution, requiring continual surveillance in order to behaviorally adapt to its changing demands (Webster and Ward, 2011). Adaptations in an individual’s behavior can also impact the social structure of the group; driving, in turn, downstream behavioral adaptations in other group members. Understanding the reciprocal interplay between individual behavior and the social environment is therefore crucial to gain insight into how individuals can be behaviorally defined, how their traits are encoded at a neural level, and how these aspects shape their responses to environmental challenges – whether social or not. However, studying fine-scale behavioral interactions in wild rodent populations is challenging (Hughey et al., 2018), considering their large territorial range and the inability to control for genetic or environmental factors (Berry and Bronson, 1992; Macdonald et al., 1999). On the other hand, containing rodents into standard laboratory housing can mask their behavioral profiles. To solve these issues, environmental enrichment can be used, which has proven to widen the set of behaviors rodents can express (Blanchard and Blanchard, 1988; Zocher et al., 2020), as well as the implementation in the laboratory of large, automated testing environments where mice or rats can live in micro-societies (groups that range in size from one to several dozen individuals) under semi-naturalistic conditions (Alexander et al., 1978; Freund et al., 2013; König et al., 2015; Torquet et al., 2018).

**FIGURE 2:**
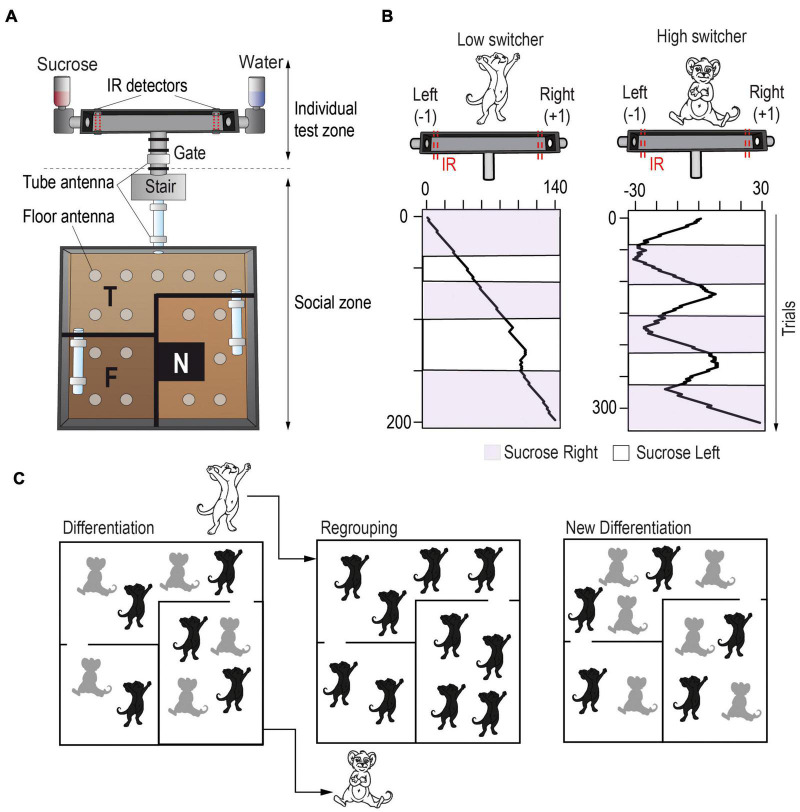
Semi-naturalistic environments allow the study of inter-individual variability with a social context. **(A)** Souris-City environment includes a large and complex living space, in which mice live together and can express sophisticated social and non-social behaviors, and an individual test zone: a T-maze delivering different drinks on each side (e.g., water or sucrose). In the T-maze, mice (inbred male C57BL/6J strain) can perform a cognitive decision-making task spontaneously and isolated from their conspecifics. The various detectors present in the environment allow to follow each individual’s behavior and estimate spontaneous individual traits. These are derived from both the general behavior expressed within the social group in the main environment, and the behavior and cognitive performance in the individual test zone. **(B)** Stable and distinctive patterns of choice strategy in the T-maze consistently emerged in independent experiments with, in particular, individuals tracking sucrose (right panel) and individuals constantly choosing the same side independently from the sucrose position (left panel). **(C)** Strikingly, when modifying the social environment by mixing mice from different Souris-City experiments but with similar behaviors, we observed a fast re-adaptation of individual traits, suggesting a social component to this individuation process (Torquet et al., 2018).

Importantly, when rodents live in micro-societies within a closed and enriched naturalistic environment, strong and stable inter-individual variability in behavior emerges, even among isogenic animals. Early studies, such as Rat Park (Alexander et al., 1978), found that rats living in complex social environments show behavioral differences from those in isolated or standard laboratory conditions, but interpretations of these initial studies are limited due to small numbers of animals and few data points (Gage and Sumnall, 2019). More recent studies using large groups of animals with automated data collection have yielded interesting results concerning inter-individual variations. For instance, when forty isogenic mice were placed in a complex environment over a period of months, significant individual differences in explorative behavior and active coverage of the territory, defined as the distribution of space that each animal occupies, were discovered (Freund et al., 2013). This spatial exploratory behavior was negatively correlated with social exploration and play behaviors estimated using manual assessment (Freund et al., 2015), while it was positively correlated with hippocampal neurogenesis (Freund et al., 2013). Our group has developed a semi-naturalistic environment called Souris-City (Figure 2A), where groups of ten mice can undergo an extended behavioral analysis over long periods of time (>1 month). Automatic capture of a large spectrum of behaviors over these longitudinal experiments demonstrates that individualistic behavioral patterns also emerge in these smaller groups of animals, with differences observed in spatial exploration and social behaviors. The Souris-City environment uses a series of RFID-sensing gates to allow the testing of individual cognitive abilities while subjects are temporarily separated from the group (Torquet et al., 2018). Thus, we can clearly distinguish between personality traits (expressed when the animal is alone) and behaviors that could be the direct consequence of a group interaction. The cognitive testing compartment of Souris-City consists of a T-maze, where each side can deliver different drinks. Mice are asked to choose between two different drinks (e.g., water and sucrose), and the position of the bottles is then inverted every three-to-four days, allowing the evaluation of their choice behavior and preferences for each subject. The position of the bottles is then inverted every three-to-four days, allowing for the evaluation of their choice behavior. Interestingly, several subgroups of stable and distinctive patterns of choice strategy consistently emerge, even though animals have low genetic diversity (Torquet et al., 2018). Some individuals systematically track the sucrose solution, while others are more likely to choose the same side of the T-maze, regardless of the drink presented (Figure 2B). These different patterns of choice strategy correlate with differences in social and spatial exploratory behavior in the main environment, and with differences in the spontaneous firing of dopaminergic neurons in reward circuits (Torquet et al., 2018). Strikingly, modifying the social environment by regrouping together individuals with a similar initial phenotype (Figure 2C) resulted in a fast re-distribution of individual traits, as well as adaptations to the firing pattern of their dopamine neurons. In other words, stable individual behavioral strategies can rapidly change in response to social challenges. This suggests that the dynamic effects of social interactions between individuals generate social specialization and reveal inter-individual differences in various, not necessarily social, behaviors.

The inter-individual variability that emerges in large environments may arise from different social regulation mechanisms. For example, dominance hierarchy within social groups is a naturally occurring and evolutionarily conserved phenomenon which readily emerges in group-housed male or female rodents (van den Berg et al., 2015; Kondrakiewicz et al., 2019). In mice, hierarchy usually develops within a few days and remains stable over weeks (Wang et al., 2011; Williamson et al., 2016). When unfamiliar mice are grouped in a tetrad, they establish a dominance hierarchy that can be analyzed by different pair contests, such as a warm spot occupancy test, territorial urine marking, or by evaluation in the tube-test where one mouse must yield to the other to exit the tube (Wang et al., 2011, 2014; Larrieu et al., 2017; Zhou et al., 2018). An important consequence of the interactions between rodents, and, in particular, of hierarchical organization, is that individuals seem to display markedly different behaviors depending on their social status. In laboratory conditions, dominant animals are more anxious (Larrieu et al., 2017), have higher social interactions, and better social memory (Battivelli et al., 2019) than subordinates. In larger groups of 10–12 mice, dominant males engage further in aggressive behavior, while subordinates modify their foraging behavior to avoid congeners by which they have previously been aggressed (Lee et al., 2018). Interestingly, in wild rodents, the configuration of colonies and overlap of territories may require an individual to act as dominant in its own territory, but perhaps as subordinate when confronted with challengers from other territories (Koolhaas et al., 1987), suggesting that hierarchical position is flexible and context-dependent. Social conflicts and aggressiveness are crucial to determine social status and access to resources, but indeed come at significant energetic cost. This leads individuals to adjust their behavior to reduce conflict, with some specializing in dominance, while others specialize in exploration and vigilance (Bergmüller and Taborsky, 2010). This process, known as “social niche specialization”, applies to all group members. It provides an adaptive explanation for the existence of hierarchy, division of labor, and individuality within a rodent group (Bergmüller and Taborsky, 2010).

The division of labor is a property that emerges in social groups. It can take different forms but mainly consists in a specialization in the execution of tasks: not everyone participates in all aspects or stages of a production process. It is an important feature of complex biological systems, particularly in social groups (Cooper and West, 2018), and it is also an active mechanism of individual differentiation (Loftus et al., 2021). Studying the division of labor at the level of resource acquisition in rodent social groups opens up very interesting perspectives for understanding the mechanisms of individuation, as it is indeed a process that allows the emergence of distinct strategies. Division of labor is well-illustrated by the observation of the coexistence within social groups of “producers” that work to search for and acquire food, and “scroungers” that subsist off of what other group members provide (Barnard and Sibly, 1981). For example, when a group of rats is placed in an apparatus where food is delivered by pressing a lever accessible to all, the “producers” press the lever while the “scroungers” simply eat the food delivered while others are pressing the lever (Oldfield-Box, 1967; Ahn et al., 2021). A similar division of labor also appears in experiments in which rats organize themselves to respond collectively to the increasing difficulty of reaching food by diving in a water-submerged corridor (Grasmuck and Desor, 2002). When in a group, some rats readily dive to fetch food, while some animals do not dive, despite successfully diving for food when alone in the apparatus, and instead they obtain their food from the others. This behavior raises the question of whether the “scroungers” are stealing food from their diving counterparts, or are these diving “producers” driven to provide for all of the members of the group, i.e., could this behavior be altruistic, or simply a process of domination? Interestingly, when the divers have the opportunity to stay in a separate place to consume the food alone, some still decide to return to the group location where the food they bring back will be eaten also by non-diving rats (Grasmuck and Desor, 2002). The proportion between “producer” and “scrounger” rats depends on the size of the group (Alfaro and Cabrera, 2021), but, overall, the repartition in each group reflects a collective behavioral balance based on contingencies between animals’ individualities and social context. Similar profiles emerge in mice when they must carry food across a pool of water; some carry the bulk of the food while others do not carry anything (Nejdi et al., 1996). These “producer” mice showed less anxiety in an elevated plus maze compared to the non-carrying “scrounger” mice, an effect interestingly seen both before the food retrieval challenge and maintained afterward – suggesting that underlying behavioral traits influence how labor is divided in social groups. Overall, four principles seem to govern these experiments: (i) taken individually, all animals are capable of solving the task, (ii) the proportion of individuals that share the same trait is related to the size of the group, (iii) individual strategy to solve the task depends on individual traits that pre-exist, and, finally, (iv) the grouping of individuals with the same profile leads to new differentiations. These elements demonstrate that variation in task performance and division of labor are social phenomena, and can be understood in terms of the equilibrium between group demand, information diffusion within the group, and individual motivations.

Large environments that embed complex tasks bring together several social processes that will cause the emergence of strong inter-individual variabilities. Dominance hierarchies and division of labor are specific examples of social niche regulation mechanisms that could help to understand the emergence of individuation, and illustrate that individual behavior is not only the result of developmental process but also of active adaptation to social challenges. Rather than the idea of a sequence of events over the course of a lifetime that drive an individual toward a phenotype, the concept of social niche specialization instead considers individual variation to be an adaptative process. Animals in large and social environments all encounter slightly different sets of life events, which gives them the opportunity to specialize in a social niche, and, in turn, results in downstream differences between individuals (Bergmüller and Taborsky, 2010; Kempermann, 2019). The development of new analytical tools, such as the continuous analysis of the animals’ poses and postures, should make it possible to now better quantify the impact of the environment and of social processes on the mechanisms of individuation and on the neurophysiological consequences of these niche specialization.

## Dopamine, a Neuromodulator at the Interface Between Social Experience and Behavioral Trait Expression

**FIGURE 3:**
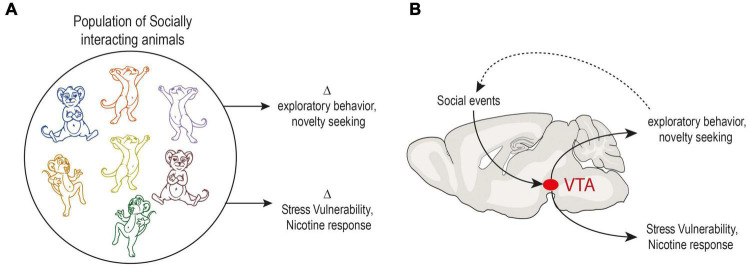
Model of behavioral adaptation in response to social context through dopaminergic system. **(A)** Traits can be viewed as stable individual expressions of a behavioral outputs or preferences – with high inter-individual variability but low intra-individual variability. In a population of animals living in a group, in the same environment, individuals adapt to their social environment. In parallel, traits emerge in a series of non-social behaviors such as exploratory behavior or novelty seeking. Differences in response to stress or drugs such as nicotine are also observed. **(B)** Diagram illustrating a possible mechanism for generating inter-individual variability. Adaptations as a consequence of individual social experience impact dopaminergic system and lead to differences in individual behavior. In turn, these behavioral differences feed forward into changes in an individual’s interactions with the environment. This loop allows a social profile to be adjusted to another behavioral profile, for example exploration level. The same type of mechanism could allow the emergence of variability in vulnerability to stress and response to drugs.

The systematic individual biases that make two individuals different imply strong constraints on neural systems. In particular, it suggests that they are in a way limited in their operating range. The study of the neural bases underlying inter-individual differences mainly focuses on two aspects: (i) pre-existing neurophysiological differences that may explain why individuals respond differently to the same experiences, and (ii) the general plasticity mechanisms that explain how neurophysiological systems adapt as a consequence of individual experience, for example in response to learning or to stress. We propose that these two aspects are coupled: adaptations as a consequence of individual experience lead to differences in individual responses. In turn, these behavioral differences feed forward into changes in an individual’s interaction with the environment. Since dopamine circuitry has been implicated in both the stability and flexibility of behaviors (Cools, 2019; Korn et al., 2021), and in various other behaviors, it is thus not only poised to play a central role in the neurophysiological mechanisms of individuation processes, but may be further conceptualized as a central mechanism of a control loop between social influences and behavioral trait expression (Figure 3).

Dopamine has long been implicated in reward, aversion, learning and motivation, as well as in various aspects of cognition (Kakade and Dayan, 2002; Schultz, 2007; Bromberg-Martin et al., 2010; Berke, 2018). But it has also been more specifically linked to a cluster of traits that appear to be strong determinants of individual personalities in rodents, including reward seeking (Ikemoto and Panksepp, 1999), novelty seeking, and exploration (Bardo et al., 1996; Kakade and Dayan, 2002; Bunzeck and Düzel, 2006). However, despite the substantial attention paid to dopamine in personality neuroscience (DeYoung, 2013), and despite the evidence pointing toward a link between modulations of dopaminergic function and variations in individual behavior, no comprehensive theory currently explains the role of dopamine in mediating individuation. Beyond the difficulty in precisely defining and measuring a trait, some elements of the physiology of dopaminergic neurons make this problem difficult to assess. Dopamine neurons show a diversity in their projection sites, receptor distribution, and patterns of firing and release; leading to a wide variety of intertwined functional and cognitive roles of dopamine signaling (Cools, 2019). They exhibit a patterned spontaneous firing activity, described as a continuum between two distinguishable rhythms: a tonic slow and regular single spike firing and a phasic bursting mode (Grace et al., 2007; Faure et al., 2014). Regular spiking emerges from intrinsic membrane potential oscillations while the burst-firing pattern critically depends on afferent networks of the dopamine neurons (Grace et al., 2007; Faure et al., 2014). Fluctuation in tonic release is associated with modulation in the firing activity of the spontaneously active population of dopamine neurons. Tonic DA release acts through the gating and modulation of the activity and input sensitivity of downstream neurons and circuits (Dayan, 2012). In contrast, phasic release is specifically associated with the synchronization of burst firing in dopamine neuron populations, and induces a substantially larger dopamine release in terminal regions (Tsai et al., 2009). Phasic dopamine provides a learning signal by encoding the difference between the expected and the actual reward, the so-called reward prediction error (RPE). Dopamine neurons increase their phasic activity first at the presentation of an unexpected reward, and then during the anticipatory phase of this reward after learning (Schultz et al., 1997). Finally, dopamine signaling also depends on clearance mechanisms, relying on the dopamine transporter in the striatum or on catechol-O-methyltransferase action in the cortex (Korn et al., 2021), which adds another layer of control and complexity. This heterogeneity in dopamine release dynamics and sites of action indeed complicates our understanding of how dopamine signaling could influence inter-individual variation and personality. Despite these difficulties, several lines of evidence suggest that variations in the basal activity of dopamine neurons and the tonic level of dopamine impact the expression of individual behavior, particularly those related to reward seeking. Further evidence indicates that dopaminergic activity is actively modulated by social behavior.

Rewards impact the organization of higher-order behaviors: they spur the construction of goals and drive the extraction of information about their presence, predictability, accessibility, and associated costs from the environment. Gathering information about uncertain rewards results in a trade-off between exploration and exploitation (Cohen et al., 2007), which is considered as one of the major axes of trait variation along with locomotor activity, boldness, aggressiveness and sociability (Gartland et al., 2021). Dopamine neuron activity is associated with the level of expression of many behavioral traits related to reward seeking, notably with the level of exploration (Cohen et al., 2007; Frank et al., 2009; Humphries et al., 2012; Schiemann et al., 2012), but also the propensity for risk taking (Onge and Floresco, 2008; Stopper et al., 2014), reaction to uncertainty (Fiorillo et al., 2003; Naudé et al., 2016) and response vigor (Niv et al., 2007). Several studies in rodents have now demonstrated the important role of tonic dopamine neuron activity in setting the balance point for the trade-off between environmental exploration and the exploitation of existing knowledge (Beeler et al., 2010; Cinotti et al., 2019; Dongelmans et al., 2021). When mice were presented with two levers in a “closed economy” paradigm where each lever had different relative costs for food, but the two levers frequently switch position, wild-type mice optimally adapted their choices by distributing more effort on the least expensive lever. Hypodopaminergic mice, however, distribute their effort roughly equally between levers expending on average more effort for each pellet earned than wild-type mice, which suggests a role for tonic dopamine in the exploration of options (Beeler et al., 2010). Antagonizing D1-D2 dopamine receptors using systemic injection of flupentixol affects the performance of rats in a 3-armed bandit task with varying levels of uncertainty, resulting in an increase in random choices. A computational analysis reveals that decreasing dopaminergic activity increases exploration, without altering learning rate (Cinotti et al., 2019). Finally, in a recent study, mice were faced with consecutive binary choices in a spatial version of the multi-armed bandit task (Figure 1B), having to choose between visiting three sites in an open field delivering an intracranial reward with different probabilities: 100, 50, or 25% (Naudé et al., 2016; Dongelmans et al., 2021). In this task, wild-type mice display individual decision-making strategies, some making more exploitative choices (visiting primarily the 100 and 50% rewarded sites), while others make more exploratory choices (incorporating information gathering about the site rewarded 25% of the time). Chronic exposure to nicotine drives mice toward more exploitative strategies, which was associated with an increase in spontaneous dopamine neuron activity (Dongelmans et al., 2021). Importantly, optogenetically mimicking the increased tonic dopaminergic activity observed under nicotine exposure is sufficient to temporarily and reversibly induce the adoption of an exploitative strategy in mice, suggesting that factors which modulate dopaminergic function can flexibly shift behavioral traits. Together, these findings show the importance of tonic dopamine release in setting the threshold between exploration and exploitation strategies, which is one crucial determinant in adaptive personality in rodents. Modifying ascending dopaminergic activity thus likely modulates arbitration between different strategies, exploiting or exploring certain options, through the gating and modulation of the downstream circuits (Dayan, 2012).

Finally, dopamine is also heavily implicated in establishing and maintaining social relationships. Vertebrate social behaviors are mainly controlled by two evolutionary conserved and interactive neural circuits (O’Connell and Hofmann, 2011): a “social behavior network” composed of midbrain, hypothalamic, and basal forebrain nuclei that is involved in aggressive, reproductive, and communication behaviors (Newman, 2017); and the reward system corresponding to the mesocorticolimbic dopamine network, that allows social behavior to be reinforcing and, thus, adaptive (O’Connell and Hofmann, 2011). Indeed, recent studies demonstrate that dopamine encodes key aspects of social interactions (Gunaydin et al., 2014), that dopaminergic reward prediction errors guide social learning (Solié et al., 2021), and that dopamine has a role in promoting aggressive behavior in mice (Golden et al., 2019; Mahadevia et al., 2021). These findings suggest that the dopaminergic system plays an essential role in social interactions by encoding information about valence (rewarding or aversive social situations), and about social positioning to drive relationship-appropriate behaviors. There is also a growing body of evidence suggesting that social experiences induce long-term modifications in spontaneous dopaminergic activity. Social defeat, an example of a negative social challenge, produces strong and long-lasting changes in spontaneous dopamine neuron activity, dopamine release within the mesolimbic dopamine pathway, and modifies social engagement, notably leading to withdrawal from social interactions (Krishnan et al., 2007; Barik et al., 2013; Chaudhury et al., 2013; Friedman et al., 2014). Social ranking in tetrads of male mice is associated with marked changes in VTA dopamine neuron activity, with higher-rank animals displaying lower bursting activity (Battivelli et al., 2019). Finally, in Souris City, we have shown that the phenotypic divergence in individual behaviors is mirrored by differences in the firing properties of midbrain dopamine neurons, and that modifying the social environment resulted in a fast re-adaptation of both the animal’s traits and the firing pattern of its dopamine neurons (Torquet et al., 2018). Stable decision-making strategies and dopaminergic neurons activity can thus rapidly change upon exposure to social challenges.

Altogether, these diverse – yet intertwined – functions of dopamine signaling suggest that this neuromodulator may link social experience with individualistic behavioral output. We propose in this review that, by triggering rapid modifications in dopaminergic function, the social environment actively alters both social and non-social behaviors, such as the trade-off between exploration and exploitation. This many-to-many relationship, where changes in regulatory influences over dopamine activity induce adaptations in multiple behaviors, has strong implications for the understanding of inter-individual variability and the link between personality, response to environmental risk factors, and mental health outcomes. Social experiences that modify dopamine function, because they would lead to a modification of a certain number of traits (e.g., the level of exploration), would make it possible (Figure 3B) to match a social profile to, for example, an exploratory profile (e.g., dominant mice explore less). A subsequent question is then to understand whether vulnerability to psychopathologies could also be extrapolated by social profiles and associated dopaminergic adaptations.

## Using Inter-Individual Variability to Predict Mental Health Outcomes

Studying the neurobiology of inter-individual variability is essential for understanding how it relates to vulnerability or resilience to psychiatric disease. Mental health disorders are highly heritable, however, their genetic risk factors account for only somewhere between 10–60% of the variance in their distribution (Kreek et al., 2005; Kendler et al., 2012; Fromer et al., 2016; Howard et al., 2019), and they result from complex polygenic interactions that can be common across multiple disorders (Kendler et al., 2003b; Pasman et al., 2018; Wray et al., 2018; Demontis et al., 2019; Linnér et al., 2021). Environmental, social, or cultural factors must therefore also play important roles in determining the incidence of psychiatric disease. Indeed, psychiatric diseases are often, but not always, incited by a precipitating environmental factor: experiencing a stressful life event, for example, has been linked with an increased risk of developing major depressive disorder (Kessler, 1997; Tennant, 2002; Kendler and Gardner, 2010); while exposure to a drug of abuse, and its subsequent availability, is a necessary environmental component for the onset of substance abuse issues (Tsuang et al., 1998; Kendler et al., 2003a; Volkow and Li, 2005). Whether inter-individual variations in behavioral trait expression interact with the social environment to shape vulnerability profiles, and the circuitry on which they may converge, are thus current topics of investigation.

Ample evidence argues that despite equal exposure to a specific psychoactive substance, not all individuals develop an addiction; just as not all individuals will develop depression after a stressful life event. An individual’s social milieu may account for this variation in the susceptibility to develop mental illness, as the quality of social relationships in adulthood significantly modulates the development of psychiatric disease, even in the face of strong environmental risk factors. Social support, in the form of healthy romantic relationships, strong familial ties, and community involvement, has been linked with a reduction in the risk of developing mental illness following stressful life events in adults (Syrotuik and D’Arcy, 1984; Kawachi and Berkman, 2001; Coker et al., 2002). Whereas negative social relationships in adulthood, including social isolation, workplace bullying, or intimate partner violence, are linked with a higher incidence of psychiatric illness (Barnett and Gotlib, 1988; Bonomi et al., 2006; Einarsen and Nielsen, 2015; Lacey et al., 2015; Verkuil et al., 2015; Rohde et al., 2016; Leigh-Hunt et al., 2017). Mental health issues, in turn, can perpetuate social isolation and/or maladaptive relationships. The notion of psychiatric vulnerability is thus tightly and bi-directionally linked to an individual’s social environment, and as such, one of the overarching consequences of our hypothesis is that a crucial aspect of vulnerability or resilience to psychopathology results from how the active adaptation of neuromodulatory networks in response to social environments constrains the cumulative effect of risk factors.

Direct causal links between an individual’s social environment and the development of mental illness are, however, challenging to establish in human populations. Nevertheless, the proximal social environment has been shown to influence the expression of depression- or addiction-like behaviors in preclinical rodent models; with negative social experience (e.g., isolation from peers, receiving repeated aggression) increasing these types of behaviors, while positive social experience (e.g., housing with peers) has been suggested to buffer the effects of stressors. While social isolation is most commonly used as a developmental stressor, isolation of adult rodents from their cagemates has been shown to promote depressive-like behaviors (Martin and Brown, 2010; Ieraci et al., 2016; Preez et al., 2020) and to increase self-administration of drugs of abuse (Alexander et al., 1978; Bozarth et al., 1989). Mice exposed to repeated aggressions in a chronic social defeat stress (CSDS) paradigm can be divided into differing phenotypes depending on the level of social avoidance exhibited following the CSDS (Kudryavtseva et al., 1991; Berton et al., 2006; Krishnan et al., 2007; Golden et al., 2011), with some mice showing marked depressive-like symptoms while others show stress resilience. Social stress in adult rodents has also been linked to increased vulnerability to develop addiction-like behaviors. Following a repeated social stress in a resident-intruder paradigm, rats showed increased conditioned place-preference to cocaine, sensitized locomotor activation in response to acute amphetamine administration, as well as increased motivation for cocaine self-administration and increased cocaine intake (Covington and Miczek, 2005; Stelly et al., 2016). Remarkably, returning rats to a positive social environment following resident-intruder stress, rather than leaving them individually housed, can counteract the enduring effects of social stress on cognitive and mood-related outcomes (Ruis et al., 1999; Frijtag et al., 2000), suggesting indeed that the interaction between social environment and stress response is bi-directional in nature and able to be modified continuously in adult rodents. Social stress is not the only factor in group interactions that can reveal individual vulnerability to behaviors linked to psychiatric disease models. The natural social milieu of a rodent, and their place within its hierarchy, can already significantly constrain, or perhaps even amplify, their reactiveness to environmental factors. Dominant mice are more susceptible to negative outcomes following CSDS (Larrieu et al., 2017) or chronic mild stress (Karamihalev et al., 2020), and to experience greater cocaine CPP (Yanovich et al., 2018) than mice lower in social ranking. Likewise, socially dominant rats showed greater cocaine intake in a self-administration experiment (Jupp et al., 2016). A major current limitation to these studies is the use of limited social groupings (using traditional rodent housing and/or single housing animals), as well as the use of acute testing to establish phenotypes. For example, to determine the susceptible vs. resilient mice following CSDS, a social interaction test of less than 5 min is typically used (Cao et al., 2010; Barik et al., 2013; Morel et al., 2017), and the mice are then divided by a median split of their interaction time. By observing mice instead in automated, semi-naturalistic environments over long periods, the characterization of how social stress affects each individual, based on their longitudinal profiles of both social and non-social behaviors, would be quantifiable as continuous variables. This approach would enable the establishment of robust correlations between behavioral trait expression (such as exploration level, Torquet et al., 2018), vulnerability profiles (e.g., by testing drug self-administration), and set the stage for unraveling the underlying circuitry.

As such, the complex neuronal circuits that underpin resilience/susceptibility profiles remain far from understood. We propose that the rapid effects of social experience on VTA dopamine neuron function influence the expression of vulnerable/resilient phenotypes with regard to depressive- or addiction-like behaviors. Individual variations in addiction vulnerability have been linked to spontaneous dopamine neuron activity; rats that show higher basal dopamine neuron firing rates and bursting activity are more likely to exhibit higher novelty or exploratory behaviors and show increased propensity to self-administer psychostimulant drugs (Piazza et al., 1989; Pierre and Vezina, 1996; Marinelli and White, 2000; Suto et al., 2001; Kabbaj, 2006; O’Connor et al., 2021). Spontaneous dopamine neuron firing is elevated following CSDS (Cao et al., 2010; Barik et al., 2013; Morel et al., 2017), an effect which is more prominent in susceptible mice than in resilient mice, as resilient mice instead actively regulate ion channels in response to this social stressor to stabilize dopaminergic cell excitability (Krishnan et al., 2007; Friedman et al., 2014, 2016). Interestingly, one of these studies also indicates that exposure to chronic nicotine, which increases dopamine neuron firing, can increase the potency of a mild social stressor, inducing a vulnerability to the negative effects of a sub-threshold social defeat (Morel et al., 2017). Furthermore, VTA nicotine receptor expression and dopamine neuron response to intravenous nicotine is altered following CSDS (Morel et al., 2017). Together, these results suggest that the modulation of dopamine firing by social defeat stress is instrumental in the development of a susceptible phenotype. Recent studies indicate that postpartum rats show transient changes in dopaminergic activity which are linked with the expression of depressive-like behaviors (Rincón-Cortés and Grace, 2019). The unique social stressor of pup removal further alters dopaminergic activity in postpartum dams, resulting in a decrease of spontaneously active dopamine neurons, which can be rescued by paring housing two pup-separated dams together (Rincón-Cortés and Grace, 2021). These results suggest provide initial evidence that social support may attenuate the effect of stressors by restoring dopamine neuron activity. Recent studies further suggest that the VTA acts as a physiological hub for determining the response to environmental stressors, since other molecular signatures of depression in humans and in rodent models are upstream of the VTA and exert their effects by altering dopamine neuron firing, including modulations in cholinergic (Small et al., 2016; Morel et al., 2017) or noradrenergic input (Isingrini et al., 2016) to the VTA. Understanding how the social environment shapes dopaminergic activity may therefore provide significant insight into individual risk profiles for developing mental health disorders.

Finally, as traits such as novelty seeking and exploration have also been linked to spontaneous dopaminergic activity level, a major open question is whether both the expression of these traits and psychiatric vulnerability share overlapping dopaminergic pathways, and would be therefore vulnerable to the same perturbations by social influence. These ideas have yet to be directly experimentally explored, as they require large experiments with mice living in micro-societies with automated data collection in order to observe correlations and test causative hypotheses. Despite these challenges, studies have recently begun to establish causality between altered dopamine function and psychiatric vulnerability or between dopamine function and level of exploration. Optogenetic experiments have shown that the direct activation or inhibition of midbrain dopamine neurons bidirectionally modulates depression-like behaviors, rescuing or augmenting susceptibility to CSDS in mice (Chaudhury et al., 2013; Tye et al., 2013). Activating or inhibiting dopamine neuron firing using optogenetics also rapidly and reversibly shifts the individualistic level of exploration behavior in a decision-making task (Dongelmans et al., 2021). Given that both dopaminergic activity and decision-making behavioral traits are indeed remodeled when faced with changing social environments (Torquet et al., 2018), whether exploration trait expression and vulnerability to depressive- or addiction-like behaviors correlate, whether they share dopaminergic pathways, and if they can be modulated in parallel by social input remains a topic of current investigation. Emerging relationships between social experience and dopaminergic function thus begin to link inter-individual variability in behavioral trait expression to the idea of an individual’s mental health trajectory. Advancing this line of research is poised to shape the future of precision psychiatry.

## Discussion

Inter-individual variability consists, in part, of the differential behavioral responses to environmental cues and challenge that define individuals, leading to what we consider to be a personality. How personalities are constructed, maintained, and changed in response to environmental challenges remains an open question. Here, we propose that social environments are major drivers of individuation processes, even in adult rodents, and even after the previously stable expression of behavioral traits. We further contend that adaptations in the activity of neuromodulatory circuits, and dopamine in particular, underlie socially-driven individuation processes. Finally, we suggest that this framework may have useful applications in understanding environmental influence on psychiatric vulnerability. We discuss throughout the important consideration for the field in the context of testing environments, as these complex socially-driven inter-individual differences are best studied in large, semi-naturalistic environments where rodents can live in groups.

Rodent micro-societies must be seen as systems, that is, as an organized set of interacting elements from which specific properties and functions emerge. In insects, mechanisms of social homeostasis (Emerson, 1956) allowing the maintenance of structures, castes, or colony’s environment (i.e., an optimal temperature in the nest for example), emerge due to asymmetries in individual environments. What is optimal for one individual is not necessarily optimal for the other. From these asymmetries emerge competition, dominance, division of labor, and marked inter-individual differences. For example, in large environments with a social component, individuals with identical genetic backgrounds are initially exposed to a seemingly identical environment. However, its perception by each individual encompasses a shared component, i.e., the context to which they are all equally exposed, as well as a non-shared component corresponding to the individual’s interpretation of environmental cues through the lens of their life history. In this non-shared environment, the social influence differs between individuals (e.g., some exert aggression toward the others, while some are subjected to it) and creates a unique experience. Individual behavioral and physiological adaptations allow the emergence of distinct and stable individual profiles (Bergmüller and Taborsky, 2010; Freund et al., 2013, 2015; Torquet et al., 2018). Thereby, behavioral traits work as a dynamic system where equilibria define stable traits. However, these traits may reorganize rapidly if environmental or physiological conditions change sufficiently. This can be seen in so-called “sociotomy” experiments, where colonies are reorganized by separating or recombining subsets of individuals. In insects, but also in rodents (Torquet et al., 2018), a rapid reconstitution of the task distribution can be demonstrated after such experiments. An interesting consequence of this point of view, still largely unexplored, is that traits associated with vulnerability to psychopathology emerge largely from environmental influence. A question that follows is whether certain environmental conditions (e.g., strong competition…) favor the emergence of these traits, how they are distributed in the population and finally whether, like division of labor, variation in trait expression emerges from social life.

A fundamental proposal in the field suggests that personality can be explained by constraints on behavioral adaptation (Sih et al., 2004; Duckworth, 2010; Wolf and Weissing, 2010). Animals can flexibly adjust their behavior over time, in response to situations. However, the fact that two individuals can be more or less aggressive compared to the average population implies a strong coherence in their behavior, and suggests that there is a limit to their respective range of adaptation. This constraint in adaptability defines an individual, gives the feeling of consistency of behavior over time and can be established and/or modified depending on the social context. We can think of these constraints as the individual being caught up in a network of reciprocal interactions between the neuronal circuits shaped by learning and the environment where the individual becomes increasingly specialized. The concepts of brain plasticity and learning thus give singularity to each individual. Brain connectivity and activity can be thought of as a dynamic system, as they control the subjective perception of the environment of an individual, and are themselves modified according to each individual’s history. Apart from those very general mechanisms, is it possible to extract the specific role of a given neural circuit in the definition of an individual? Here, we propose that the dopaminergic system, at the interface between adaptation, neuromodulation and decision making, plays a particular role in the control of interindividual differences.

While studies categorizing rodents based on locomotor or social stress-related behaviors provide an entry into the relationship between dopamine neurophysiology, social behavior, and psychiatric vulnerability, they fall prey to some of the same caveats with minimizing interindividual variability in experimental conceptualization. Creating categorical variables from a continuous distribution indeed simplifies data analysis and presentation, however, such an artificial creation of distinct groups can result in a loss of information from the original continuous dataset and a significant limitation of the predictive validity of the variable in question. For example, a categorical value derived from a median split will represent equally those values closest to and those farthest from the median within each group, sacrificing inter-individual variability within the group(s) and thus reducing the power of predictive analyses that can be made using regression (DeCoster et al., 2010). The artificial categorization of continuous distributions indeed significantly facilitates statistical calculations, allowing means comparisons where regression would be more appropriate. Nowadays, with rapidly expanding computational properties, the ability to easily assess nuanced relationships between behavioral distributions and physiological markers is now feasible. The use of large and automated testing environments therefore represents an enormous advantage in predicting psychiatric vulnerability from behavioral or physiological traits, as they are able to measure multiple continuously distributed variables per subject. Thus, while emerging relationships between social experience and dopaminergic function can be linked to the idea of an individual’s mental health trajectory, our knowledge to date of the relationships between these factors and psychiatric outcomes remains limited. We propose that the proximal social environment limits the adaptability of neuromodulatory networks when faced with triggering events for the emergence of psychiatric disease, thus constraining an individual into a more susceptible or more resilient state. This state can be thus predicted from the expression of particular behavioral traits. Notably, this theory, and the results supporting it to date, strongly suggest that positive social connections are a key environmental intervention to support equilibrated mental health. The advancement of trait assessment in large automated testing environments will drive this line of research forward in an unbiased and accurate manner.

## Author Contributions

All authors listed have made a substantial, direct, and intellectual contribution to the work, and approved it for publication.

## Conflict of Interest

The authors declare that the research was conducted in the absence of any commercial or financial relationships that could be construed as a potential conflict of interest.

## Publisher’s Note

All claims expressed in this article are solely those of the authors and do not necessarily represent those of their affiliated organizations, or those of the publisher, the editors and the reviewers. Any product that may be evaluated in this article, or claim that may be made by its manufacturer, is not guaranteed or endorsed by the publisher.
